# Obese mother, obese child: A convergent mixed methods study of intergenerational obesity in low-income households in Malaysia

**DOI:** 10.1371/journal.pone.0355312

**Published:** 2026-08-11

**Authors:** Nur Nadia Mohamed, Abdul Jalil Rohana, Noor Aman A Hamid, Frank B Hu, Vasanti S Malik, Muhammad Fadhli Mohd Yusoff

**Affiliations:** 1 Dietetics Programme, School of Health Sciences, Universiti Sains Malaysia, Kubang Kerian, Kelantan, Malaysia; 2 Department of Community Medicine, School of Medical Sciences, Universiti Sains Malaysia, Kubang Kerian, Kelantan, Malaysia; 3 Department of Nutrition, Harvard T.H. Chan, School of Public Health, Boston, Massachusetts, United States of America; 4 Department of Nutritional Sciences, Faculty of Medicine, University of Toronto, Toronto, Canada; 5 Institute for Public Health, Ministry of Health Malaysia, Shah Alam, Selangor, Malaysia; Lusofona University of Humanities and Technologies: Universidade Lusofona de Humanidades e Tecnologias, PORTUGAL

## Abstract

Maternal and child overweight and/or obesity (OW/OB) are growing public health concerns, especially in low-income households, where they contribute to intergenerational transmission and increased financial burden. Using a convergent mixed methods design, this study aimed to investigate the determinants of intergenerational obesity among low-income families in Malaysia. Quantitative data were derived from the Malaysian National Health and Morbidity Survey for the years 2006, 2011, and 2015, while qualitative insights were obtained through in-depth interviews. The quantitative study included 2,057, 994, and 952 mother-child pairs from low-income households in 2006, 2011, and 2015, respectively classified based on body mass index categories. Factors associated with overweight mother/overweight child pairs (OWM/OWC) were analyzed using multiple logistic regressions. To explore the factors contributing to OW/OB, the qualitative study involved 27 in-depth interviews with mothers, and the data were analyzed using thematic analysis and findings from both studies were integrated through a narrative approach. Maternal ages between 41–50 years (2006: AOR = 2.35, 95% CI = 1.21–4.60, p = 0.012) and above 50 years (2006: AOR = 2.29, 95% CI = 1.06–4.98, p = 0.036; 2015: AOR = 3.23, 95% CI = 1.14–9.13, p = 0.025) were associated with higher odds of OWM/OWC. Children aged between 10–14 years had higher risk (2006: AOR = 2.38, 95% CI = 1.63–3.47, p < 0.001; 2011: AOR = 1.76, 95% CI = 1.05–2.96, p = 0.032). Chinese (2006: AOR = 0.46, 95% CI = 0.23–0.92, p = 0.027; 2015: AOR = 0.40, 95% CI = 0.19–0.83, p = 0.014), other ethnicities (2006: AOR = 0.49, 95% CI = 0.32–0.73, p = 0.001; 2011: AOR = 0.46, 95% CI = 0.25–0.83, p = 0.010; 2015: AOR = 0.53, 95% CI = 0.31–0.91, p = 0.020), and large household size (2015: AOR = 0.19, 95% CI = 0.04–0.94, p = 0.041) were protective against OWM/OWC. Four major themes emerged from the in-depth interviews: (1) personal factors (demographics, cognitions, and skills), (2) social environment (parent-child relationships), (3) physical environment (home and built environments), and (4) macro-level environment (food price and media influence). The findings suggest that intergenerational OW/OB in low-income households is influenced by interconnected personal factors, as well as social, physical, and macro-level environments. This study not only provides a comprehensive understanding of how such factors interact within real-life contexts but also highlights the importance of multi-level interventions that address individual behaviors and broader environmental and socio-economic constraints.

## Introduction

The prevalence of overweight and/or obesity (OW/OB) has increased worldwide, with the highest rates observed among women [1]. Additionally, women with OW/OB tend to have children with similar problems, resulting in intergenerational OW/OB [2]. This phenomenon has been associated with substantial health consequences across the life course [3].

OW/OB has placed substantial financial burdens on the healthcare systems of many countries [4]including Malaysia, which the highest related healthcare costs in Southeast Asia [5]. OW/OB also imposes considerable economic burdens on families. For instance, a previous study revealed that mothers with OW/OB spent a higher health care expenditures on their children than normal-weight mothers [6].

In the past, the problem of OW/OB in low- and middle-income countries (LMICs) was common among individuals from high socioeconomic status, while those from low socioeconomic groups typically suffered from undernutrition [7]. However, the socio-economic transition experienced in LMICs has shifted the trend, such that OW/OB is now more prevalent in lower socioeconomic groups [8]. For this reason, it is essential to understand how intergenerational OW/OB occurs in low socioeconomic groups. A few studies have investigated low-income households to explore the perceptions and experiences of mothers and their children in relation to how they become overweight or obese [9–11]. However, these studies were conducted only in high-income countries.

At present, environmental and behavioural factors are increasingly recognized as important contributors to OW/OB [12]. The interrelationship between personal and multilevel environmental determinants of OW/OB can be understood using socio-ecological models [13,14]. Furthermore, the application of social cognitive theory in OW/OB research can help in understanding the possible interactions between individual factors, behaviours, and environments [15].

As the cycle of intergenerational OW/OB continues to the next generation, there is an urgent need to address this problem by investigating its determinants using quantitative and qualitative approaches. Although previous studies have examined obesity and related risk factors among adults and children, few have integrated quantitative and qualitative approaches to investigate intergenerational obesity among mother-child pairs, particularly in low-income households in the Malaysian context. To date, most obesity-related studies in Malaysia have primarily used quantitative approaches, with only a few using qualitative designs [16–18].

In the current study, the quantitative component identified socio-demographic factors associated with intergenerational obesity, including maternal age, child age, ethnicity, and household size. However, it is unable to explain the behavioural, familial, and environmental factors that contribute to the coexistence of OW/OB among mothers and children. Therefore, the qualitative component was included to explore how other factors such as household food preparation practices, parental modelling, family routines, food purchasing decisions, neighbourhood environments, and economic constraints, influence intergenerational OW/OB among low-income households.

The contributions of the current study hinge on its use of a convergent mixed methods design, which integrates nationally representative survey data with qualitative exploration of mothers’ lived experiences. This integrated approach provides a more comprehensive understanding of the socio-demographic, behavioral, social, and environmental determinants of intergenerational obesity among low-income households in Malaysia. Therefore, this study was conducted to explore the determinants of intergenerational OW/OB in low-income households in Malaysia.

## Materials and methods

### Study design

**Fig 1:**
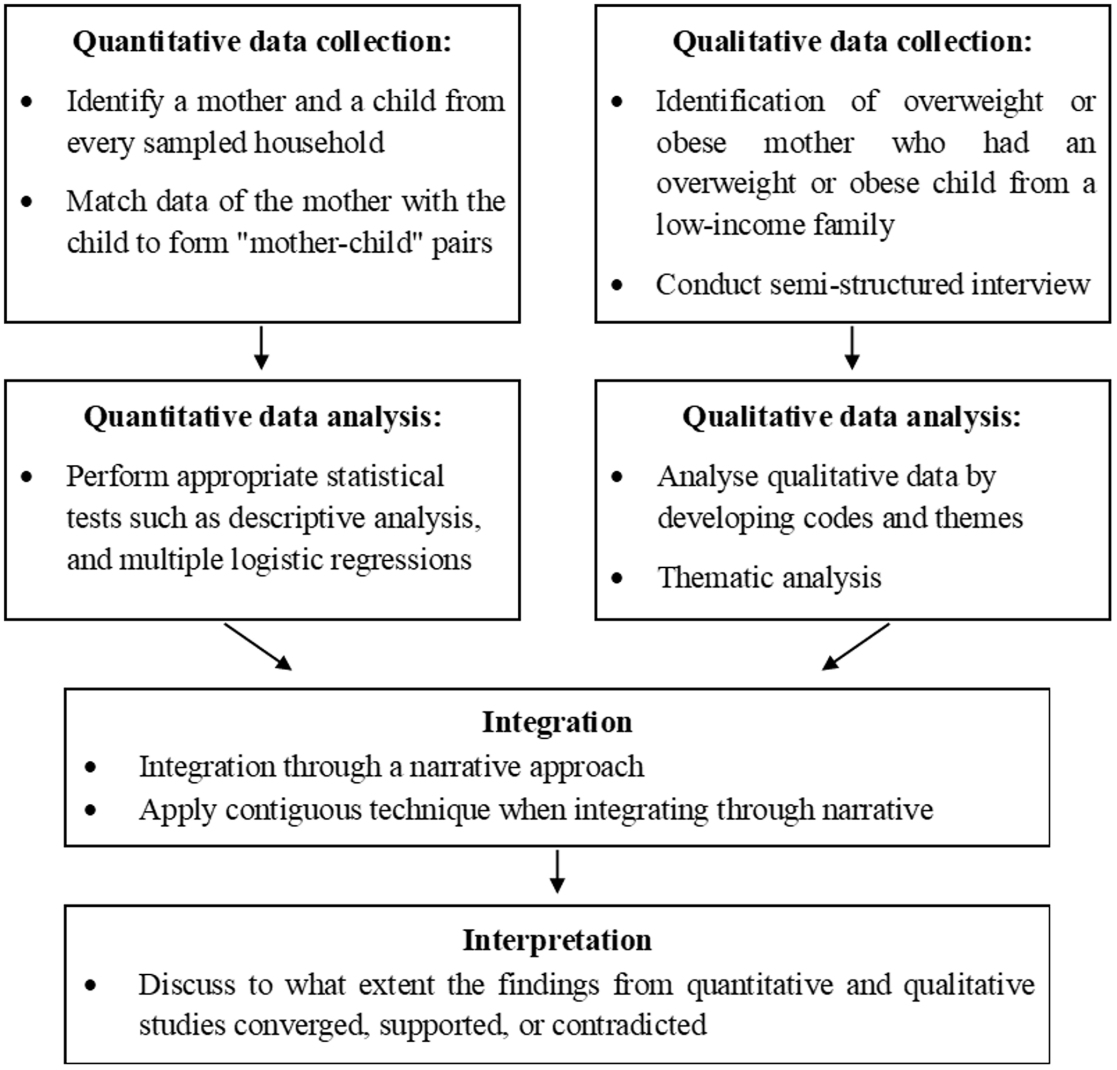
Diagram of the convergent mixed methods design, adapted from Creswell and Clark (2017).

In this study, a convergent mixed methods design [19] was applied. This was chosen because a quantitative or qualitative method alone is insufficient to demonstrate the complex and multifactorial influences of OW/OB. In a convergent mixed methods design, quantitative and qualitative data were collected and analyzed independently. Detailed quantitative methods have been reported previously [20]. In this paper, we present the process and findings of the qualitative study and integrate the findings of both studies through a narrative in the discussions [21]. The overall process of the convergent mixed method design is illustrated in Fig 1.

### Ethical approval

Ethical approval to conduct this study was obtained from the Medical Research Ethics Committee, Ministry of Health Malaysia (NMRR-17-2714-38075), and the Human Research Ethics Committee of Universiti Sains Malaysia (USM/JEPeM/17110579). Approval to use the Malaysian National Health and Morbidity Survey (NHMS) data for the quantitative study was acquired from the Director General of Health Malaysia.

### Quantitative study

#### Study participants.

The participants in the quantitative study were identified and selected from NHMS 2006, 2011, and 2015 databases. Within each household, eligible mother-child pairs were identified manually from anonymized datasets, using information on household identification variables, relationship to the head of the household, sex, and age to establish biologically plausible mother-child pairs. Only one eligible mother-child pair was selected from each household on the basis of the predefined inclusion criteria. The selection of variables was guided by theoretical relevance and previous literature on intergenerational obesity [22]. The mothers were divided into three groups in accordance with their body mass index (BMI) categories [23]: underweight (< 18.5 kg/m²), normal weight (18.5–24.9 kg/m²) and overweight (25 kg/m² and above), while their children were also categorized into three groups: underweight (BMI z-score < –2SD), normal weight (BMI z-score between –2SD to +1SD) and overweight (BMI z-score > +1SD) [24]. Then, they were matched in accordance with their BMI categories. Only mother-child pairs from low-income households were included in this study. The final quantitative sample consisted of 2,057, 994, and 952 pairs from the NHMS 2006, 2011, and 2015, respectively, after applying the inclusion and exclusion criteria. Detailed information on data cleaning and the formation of mother-child pairs has been published elsewhere [20].

#### Data analysis of quantitative study.

The analysis of quantitative data was conducted using SPSS, version 26 (IBM, Chicago, IL, USA). Simple and multiple logistic regression analyses were performed to determine the factors associated with OW/OB among Malaysian mother-child pairs in low-income households. The dependent variable in the logistic regression analyses was overweight mother/overweight child pairs (OWM/OWC) with normal weight mother/normal weight child pair (NWM/NWC) as the reference group. Independent variables included maternal age, child age, ethnicity, maternal education level, household size, and other socio-demographic characteristics. Variables with p < 0.25 in simple logistic regression analyses and variables considered clinically or theoretically relevant based on previous literature were entered into the multiple logistic regression models [25]. The model fitness was checked using the Hosmer-Lemeshow goodness-of-fit test, classification table, and area under the Receiver Operating Characteristic (ROC) curve. Findings at p < 0.05 was considered statistically significant. A detailed data analysis procedure conducted in the quantitative study was reported previously [20].

### Qualitative study

#### Study settings.

In the present study, the states were selected purposefully from the East Coast (Kelantan), Northern (Kedah, Penang, and Perak), and Central (Selangor and Federal Territory of Kuala Lumpur) regions due to practicality. Data collection was not conducted in the Southern region, Sarawak, and Sabah because data saturation had already been reached.

Participant recruitment was conducted in urban and rural areas because people from low socioeconomic groups in these living areas might have different experiences that caused them to experience OW/OB. However, the vicinity of either rural or urban area was not determined as this was not the research question that needed to be addressed. Based on the literature appraisal, obesity is rampant regardless of whether individuals live in rural or urban location. Furthermore, data in the quantitative study were analyzed in the initial stage regardless of whether the participants were in rural or urban areas.

#### Recruitment and data collection.

Recruitment and data collection for the qualitative study began on 1/10/2018 until 30/4/2019. In this study, only women from low-income households with a BMI of at least 25 kg/m^2^ and above [23] and have an OW/OB child (BMI-for-age z-score above +1 SD) aged between 5–17 years were interviewed. Here, a ”low-income household” is defined as being in the bottom 40% (B40) of the income distribution in Malaysia or having a total monthly household income of less than RM 3,860 [26]. Pregnant women were excluded because their anthropometric measurements increased rapidly during gestation.

The qualitative interviews were conducted among Malay mothers because Malays represented the majority ethnic group within the recruited low-income communities and shared relatively similar cultural and linguistic backgrounds that facilitated an in-depth exploration of their lived experiences. The methodologies involved in identifying potential participants, recruitment, and data collection have been described elsewhere [27].

For the sake of ensuring cultural appropriateness and minimal-risk study procedures, verbal informed consent was obtained from all participants prior to data collection. The consent process involved explaining the study objectives, procedures, voluntary nature of participation, confidentiality, and participants’ right to withdraw at any time without consequences.

In-depth interviews were conducted in the Malay language and the dialect of the participants. Using the regional dialect during the interviews helped the participants feel comfortable and enable them to express themselves freely when answering the questions. Only mothers were interviewed because they were usually involved in food purchasing and preparation [28]. Additionally, the study specifically aimed to explore maternal perspectives regarding intergenerational obesity and to maintain consistency in the source of information across the participants.

The participants were recruited using snowball sampling, whereby the interviewed mothers recommended other eligible participants from similar low-income communities. Although this approach facilitated access to more participants with relevant lived experiences, it may have introduced selection bias because they may have referred individuals with similar social backgrounds, experiences, or perspectives.

Semi-structured interview guides (Table 1) were employed during interviews to ensure consistency in the participants’ responses. Probing questions that began with “why”, “how”, and “can you explain more about that?” were used to gain more information from the mothers. These probes explored how mothers perceived parental influences on children’s eating and physical activity behaviors, family food preparation practices, modelling of dietary habits, feeding strategies, household routines, and environmental factors influencing both maternal and child weight status. Examples of probing questions included: “How are food-related decisions made in your household?”, and “How does your daily routine affect your eating patterns and physical activities and those of your child?” Field notes were documented during the interviews to support contextual data interpretation. The same researcher performed the interview session, which lasted from 50 to 60 minutes on average. For the next interview, the mothers were asked to recommend other mothers who met the criteria to participate in this study and could share their experiences related to the research question. Each participant was given a small token of appreciation at the end of the data collection.

**Table 1:** Semi-structured interview guides.

Item	Questions
1.	Please tell me about your daily food intake.
2.	Please tell me about your daily physical activities.
3.	What do you think about your child’s current body weight?
4.	Please tell me about your child’s daily food intake.
5.	Please tell me about your child’s daily physical activities.
6.	Why do you think both you and your child are overweight/obese?
7.	How are food decisions made in your household, and who usually influences them?
8.	How do you think your eating habits influence your child’s eating behaviors?

#### Data analysis of the qualitative study.

Data analysis was conducted simultaneously with data collection until data saturation had been met, a point when no new codes, themes, or relevant conceptual insights emerged from the interviews. Data saturation was assessed continuously throughout the data collection and analysis by comparing newly generated codes with previously identified themes and evaluating whether additional interviews contributed meaningful new information related to the research objectives. In this study, saturation was first observed after 23 interviews, after which four subsequent interviews were conducted to confirm saturation. As these additional interviews yielded no substantially new information, data collection was concluded after a total of 27 interviews.

Every interview was transcribed verbatim and coded before the next interview to ensure that the theme saturation had been met. Data were coded manually using thematic analysis procedures. Manual coding was preferred over electronic coding because of the small number of participants [29] and because it helped the researchers familiarize themselves with the collected data [30].

The qualitative data were analyzed using thematic analysis [30]. First, after the interviews were transcribed verbatim based on the participants’ dialects, the transcripts were read and re-read by the researchers to familiarize themselves with the data. Notes and ideas for coding were written on the transcripts. Next, initial codes were generated from the transcripts. Highlighters and coloured pens were used to write notes and codes on the hardcopy printouts. The research question, objective, and theoretical framework of this study were referred to as guides in focusing and deciding on the codes. Again, every transcript was read a few times and given as many codes as possible to indicate the ideas or concepts contained therein. The transcript was transcribed and coded before conducting the following interview with other participants. This process was completed until data saturation was reached. Repetition of participants’ responses across the major thematic categories and consistency in the emerging patterns indicated that thematic saturation had been achieved.

After completing the coding process, the codes were organized into potential themes using tables and mind maps. A thematic map was developed to determine the connection between the codes and themes. Not all codes were used to develop themes; some were used as sub-themes, while others were refined or discarded. The themes were then reviewed by reading all the coded data extracts and the whole data set. A thematic map was developed from the analysis, after which every theme was refined with a clear definition, and names were generated. Finally, the findings from the thematic analysis were reported. A codebook was kept during the data analysis to record emerging codes. This was developed based on the research question, literature review, and theoretical frameworks of social cognitive theory and socio-ecological model of OW/OB.

The rigor or trustworthiness of the present study was evaluated using four strategies; credibility, transferability, dependability, and confirmability [31]. In particular, this study employed member checking, peer debriefing, referential adequacy, and triangulation to ensure credibility. During member checking, four participants were chosen randomly and contacted again to check the transcripts. They were asked whether the data coded and interpreted by the researcher matched their thoughts. All the participants agreed with the researcher’s interpretation.

Next, the resulting the interview transcripts, codes, and themes were extensively reviewed by two debriefers, the individuals who had knowledge of the interview subject and the methodological issues. One of the debriefers was a content expert with a nutrition background, and the other was a qualitative research expert. After several peer debriefing sessions, both debriefers agreed with all the codes and themes.

Another strategy used to ensure the credibility of this study is referential adequacy in which the interview quotes were attached to the emerging themes. The conversations between the interviewer and the participants were audio-recorded to provide referential adequacy. Furthermore, credibility was enhanced via data and investigator triangulation. Specifically, the data were triangulated using participant interviews and field observation, while investigator triangulation was achieved by involving both academic supervisors, who were also research team members to read, review, and code the transcripts.

The strategies to obtain transferability involved using purposive sampling and providing thick descriptions of the study context. Here, the study participants were purposely selected among OW/OB mothers from low-income households to share their experiences regarding the study topic. Thick descriptions of the participants’ characteristics, the data collection process, duration, and location of the data collection were all described.

In this study, dependability was established through the use of audit trails. In particular, field notes and memos used in the interview session, as well as observations describing the locations and communities within the study areas, were kept to ensure trustworthiness. The interview transcripts were also readily available to other researchers. Additionally, the procedure was recorded for checking by the research teams to achieve confirmability.

### Integration of quantitative and qualitative findings

The quantitative and qualitative study findings were integrated at the interpretation level through a narrative technique in which, both findings were presented in a single or series of reports [32]. A contiguous approach was applied when integrating the findings. In this approach, the results from the quantitative study were reported first, followed by the qualitative study findings.

## Results

### Quantitative results

The findings of the quantitative study were published earlier [20]. Table 2 presents the characteristics and BMI categories of the mother-child pairs from the quantitative study. Many of the mothers were aged between 31–40 years (2011 = 39.4%; 2015 = 38.9%) and 41–50 years (2006 = 36.4%). Most of them were Malay (2006 = 63.4%; 2011 = 62.1%; 2015 = 63.6%), lived in rural area (2006 = 64.8%; 2011 = 57.8%; 2015 = 57.7%) and had completed primary (2006 = 42.2%) and secondary (2011 = 54.9%; 2015 = 59.5%) educational levels.

**Table 2:** Characteristics and BMI categories of mother-child pairs from low-income households in the quantitative study.

Characteristics of mother-child pairs	2006		2011		2015	
	(n=2,057)		(n=994)		(n=952)	
	n	%	n	%	n	%
Maternal age in years						
< 30	241	11.7	92	9.3	94	9.9
31 – 40	749	36.4	392	39.4	370	38.9
41 – 50	781	38.0	384	38.6	346	36.3
51 and above	286	13.9	126	12.7	142	14.9
Child age in years						
5 – 9	1249	60.7	559	56.2	545	57.2
10 – 14	581	28.2	285	28.7	273	28.7
15 – 17	227	11.0	150	15.1	134	14.1
Gender of child						
Girl	971	47.2	500	50.3	480	50.4
Boy	1086	52.8	494	49.7	472	49.6
Maternal education level						
Tertiary	10	0.5	48	4.8	68	7.2
Secondary	834	40.7	544	54.9	563	59.5
Primary	865	42.2	308	31.1	262	27.7
None	340	16.6	90	9.1	54	5.7
Ethnicity						
Malay	1304	63.4	617	62.1	605	63.6
Chinese	119	5.8	76	7.6	80	8.4
Indian	115	5.6	58	5.8	54	5.7
Other^a^	519	25.2	243	24.4	213	22.4
Household size						
Small	868	42.2	430	43.3	525	55.1
Medium	980	47.6	492	49.5	394	41.4
Large	209	10.2	72	7.2	33	3.5
Family structure						
Dual-parent family	1869	90.9	898	90.3	843	88.6
Single-parent family	188	9.1	96	9.7	109	11.4
Residential area						
Rural	1332	64.8	575	57.8	549	57.7
Urban	725	35.2	419	42.2	403	42.3
BMI categories of mother-child pairs						
UWM/UWC	18	0.9	9	0.9	6	0.6
UWM/NWC	60	2.9	22	2.2	25	2.6
UWM/OWC	10	0.5	3	0.3	2	0.2
NWM/UWC	123	6.0	46	4.6	34	3.6
NWM/NWC	620	30.1	232	23.3	221	23.2
NWM/OWC	102	5.0	54	5.4	52	5.5
OWM/UWC	73	3.5	48	4.8	37	3.9
OWM/NWC	811	39.4	430	43.3	378	39.7
OWM/OWC	240	11.7	150	15.1	197	20.7

SD = standard deviation; UWM/UWM = underweight mother/underweight child; UWM/NWC = underweight mother/normal weight child; UWM/OWC = underweight mother/overweight child; NWM/UWC = normal weight mother/underweight child; NWM/NWC = normal weight mother/normal weight child; NWM/OWC = normal weight mother/overweight child; OWM/UWC = overweight mother/underweight child; OWM/NWC = overweight mother/normal weight child; OWM/OWC = overweight mother/overweight child.

^a^Small = less than five persons; medium = 5–7 persons; large = more than seven persons in the household.

^b^Other = Other three major ethnic groups in Malaysia, including Bumiputras in Sabah and Sarawak.

Most of the children were aged between 5–9 years (2006 = 60.7%; 2011 = 56.2%; 2015 = 57.2%) and lived in dual-parent families (2006 = 90.9%; 201 = 90.3%; 2015 = 88.6%). The proportions of boys and girls were almost similar in each sample year.

Based on the BMI categories of mother-child pairs, the proportion of OWM/OWC in 2006 was 11.7%, which was double that in 2015 (20.7%). Meanwhile, the proportion of NWM/NWC from low-income households was 30.1% in 2006 and decreased to 23.2% in 2015.

The results of the univariable logistic regression for the factors associated with OWM/OWC from low-income households in the quantitative study are reported in S1 Table. Table 3 presents the final multivariable logistic regression analysis for the factors associated with OWM/OWC from low-income Malaysian households for 2006, 2011, and 2015. The results showed that the risk of OW/OB mother-child pairs in low-income households was higher among mothers aged between 41–50 years (2006: AOR = 2.35, 95% CI = 1.21–4.60, p = 0.012) and above 50 years (2006: AOR = 2.29, 95% CI = 1.06–4.98, p = 0.036; 2015: AOR = 3.23, 95% CI = 1.14–9.13, p = 0.025). Among children, those aged between 10–14 years had higher odds of being in OW/OB mother-child pairs (2006: AOR = 2.38, 95% CI = 1.63–3.47, p < 0.001; 2011: AOR = 1.76, 95% CI = 1.05–2.96, p = 0.032). Ethnicity was also significantly associated with intergenerational obesity, with Chinese (2006: AOR = 0.46, 95% CI = 0.23–0.92, p = 0.027; 2015: AOR = 0.40, 95% CI = 0.19–0.83, p = 0.014) and Other (2006: AOR = 0.49, 95% CI = 0.32–0.73, p = 0.001; 2011: AOR = 0.46, 95% CI = 0.25–0.83, p = 0.010; 2015: AOR = 0.53, 95% CI = 0.31–0.91, p = 0.020) ethnicities having lower odds of OWM/OWC compared with Malays. A large household size (2015: AOR = 0.19, 95% CI = 0.04–0.94, p = 0.041) was also protective against OWM/OWC.

**Table 3:** Multivariable logistic regression analysis for the factors associated with overweight mother/overweight child pairs from low-income Malaysian households in 2006, 2011 and 2015.

Risk factors	2006 (n=860)				2011 (n=384)				2015 (n=418)			
	AOR	95% CI		p-value	AOR	95% CI		p-value	AOR	95% CI		p-value
Maternal age												
≤ 30	1.00				1.00				1.00			
31 – 40	1.66	0.86, 3.18		0.130	0.91	0.39, 2.11		0.819	1.40	0.65, 3.04		0.395
41 – 50	**2.35**	**1.21, 4.60**		**0.012**	1.04	0.44, 2.49		0.929	1.70	0.74, 3.89		0.194
51 and above	**2.29**	**1.06, 4.98**		**0.036**	2.06	0.68, 6.25		0.201	**3.23**	**1.14, 9.13**		**0.025**
Child age												
5 – 9	1.00				1.00				1.00			
10 – 14	**2.38**	**1.63, 3.47**		**<0.001**	**1.76**	**1.05, 2.96**		**0.032**	1.45	0.85, 2.49		0.174
15 – 17	1.76	0.99, 3.10		0.052	0.81	0.36, 1.84		0.612	0.59	0.28, 1.23		0.158
Household sizeª												
Small	1.00				1.00				1.00			
Medium	0.75	0.53, 1.06		0.106	0.68	0.42, 1.09		0.106	1.01	0.64, 1.60		0.963
Large	0.60	0.33, 1.10		0.096	0.55	0.21, 1.47		0.234	**0.19**	**0.04, 0.94**		**0.041**
Ethnicity												
Malay	1.00				1.00				1.00			
Chinese	**0.46**	**0.23, 0.92**		**0.027**	0.46	0.21, 1.03		0.058	**0.40**	**0.19, 0.83**		**0.014**
Indian	1.51	0.74, 3.08		0.259	2.29	0.94, 5.55		0.068	1.24	0.56, 2.73		0.596
Other^b^	**0.49**	**0.32, 0.73**		**0.001**	**0.46**	**0.25, 0.83**		**0.010**	**0.53**	**0.31, 0.91**		**0.020**

*Note.* CI=confidence interval; AOR=adjusted odds ratio.

Statistically significant p values (p < 0.05) are highlighted in bold.

No multicollinearity and interaction was detected.

Hosmer-Lemeshow test (2006: p=0.160; 2011: p=0.644; 2015: p=0.561).

Area under the ROC curve (2006=69.1%; 2011=66.3%; 2015=63.8%).

^a^Small=less than five persons; medium=5 to 7 persons; large=more than seven persons in the household.

^b^Other=Other three major ethnic groups in Malaysia, including Bumiputras in Sabah and Sarawak

The quantitative study’s findings indicate that socio-demographic characteristics play an important role in intergenerational obesity among low-income households, thus providing a foundation for understanding the behavioral and environmental influences explored in the qualitative findings. These findings were further explored in the qualitative interviews to understand how household practices, social relationships, and environmental constraints may explain the observed statistical associations.

### Qualitative findings

A total of 27 mothers aged between 27 and 52 years, were recruited to participate in the in-depth interviews. Of these, 17 had completed secondary education, six had completed primary education, and four had a tertiary education level. One-third (n = 9) were homemakers, while the others were employed in various sectors. The number of children in the family ranged from one to seven. Most mothers lived with their husbands (n = 22), except three: one lived only with her children because her husband worked away from home, while the other two mothers were widows.

The determinants of intergenerational OW/OB among mother-child pairs in Malaysian low socioeconomic households were captured in four major themes: 1) personal factors, 2) social environment, 3) physical environment, and 4) macro-level environmental factors. The themes and sub-themes identified from the interviews are summarized in Table 4.

**Table 4:** Summary of themes and sub-themes that emerged from the interviews.

Themes	Sub-themes
1. Personal factors	1.1 Cognitions 1.2 Demographics 1.3 Skill
2. Social environment	1.4 Mother-child relationship 1.5 Paternal influence
3. Physical environment	1.6 Home food environment 1.7 Built environment
4. Macro-level environment	1.8 Food price 1.9 Media influence

The findings demonstrated that intergenerational obesity was not driven by individual behaviors alone, but rather by the interactions between personal, familial, environmental, and structural influences that collectively shaped food choices, eating patterns, and physical activity behaviors within low-income households. The qualitative findings generally converged with the quantitative results, particularly in terms of explaining the patterns observed among Malay low-income households, comprising the qualitative sample and representing the majority ethnic group in the study context. However, this convergence should be interpreted with caution, as the qualitative findings may not fully explain the quantitative associations observed among Chinese, Indian, and other ethnic groups whose sociocultural and environmental contexts may differ.

#### Theme 1: Personal factors.

In the main theme of personal factors, three sub-themes were attributed to intergenerational OW/OB among mother-child pairs: 1) cognition, 2) demographic, and 3) skill.

##### Sub-theme 1.1: Cognition:

Nearly all mothers described themselves as the primary food preparers within the household, although the extent of control over food choices varied based on their children’s preferences and household circumstances. Aside from food preparation, the mothers also stated that their children’s food preferences influenced their decisions about what food products to purchase during grocery shopping. They continually allowed their children to choose which food they liked. However, their children often choose junk food over healthy food. If the mothers made choices, the children often rejected the food they prepared due to unfavorable tastes, among others. This was explained by Mrs. CY;

*“She (her child) likes biscuits with cheese. She chooses it because she is going to eat it later. Sometimes she refuses to eat if I make the choice. I ask her to choose which one she wants to eat. So, I am not going to make the selection on her behalf. She refused to eat it when I bought it for her. She is not interested. She said that it is not tasty.”* (Mrs. CY, 27 years, homemaker)

In some families, the mothers admitted that they consumed all the foods rejected by their children because they did not want them to be wasted. They were also aware that they gained weight because they constantly consumed these leftover foods, a sentiment illustrated by Mrs. KN;

*“She does not eat (the food that she dislikes). She only eats the outer skin. After that, she leaves the food. Her mother would eat. That is why her mother is ‘skinny’.”* (Mrs. KN, 45 years, food seller)

Several mothers admitted that they rarely prepared food because they lacked motivation to prepare meals at home. Instead, they preferred to purchase takeaway food or eat at food outlets with their children. As shared by Mrs. LB;

*“I buy ‘pattaya’ fried rice (fried rice covered in a thin fried egg). Sometimes, I buy fried vermicelli. I am too lazy to cook. So I just buy it. Sometimes, I buy vermicelli soup. That’s all. I bought a pack for everyone.”* (Mrs. LB, 52 years, food seller)

These views were commonly expressed among both homemakers and employed mothers, suggesting that children had a widespread influence on household food decisions across different family situations. Mothers experiencing financial hardship also more frequently described consuming leftover foods to avoid food wastage.

##### Sub-theme 1.2: Demographics:

Most of the mothers stated that the number of individuals in the household influences food preparation at home. On the one hand, mothers from smaller households more frequently described takeaway foods as practical and economical. They commented that every time they cooked for their families, they ended up wasting food because only a small number of family members ate them. Mrs. KS, a mother of five children, rarely cooked food at home because she only lived with her two children; the other children worked in different districts, so they stayed in hostels. Thus, Mrs. KS thought that purchasing takeaway food was more economical than cooking food at home, as reflected in this quote:

*“Sometimes, we cannot finish eating a handful of rice. We throw it away and feed it to the chickens. Therefore, I do not cook. There is no need to cook. If we eat, it will cost us five ringgits for two people. The others are at work. She goes to school. Her sister is working. Only two people are left at home.”* (Mrs. KS, 46 years, food seller)

On the other hand, mothers from larger households commonly preferred preparing meals at home because purchasing takeaway foods for many family members was perceived as financially burdensome. Some mothers, including Mrs. CY, believed that purchasing takeaway food or eating out was more expensive than home food preparation. Mrs CY is a homemaker with three children; she also lives with her husband, mother, and elder brother. She stated at preference for preparing food at home because it was more cost-effective. She added that home food preparation allowed her to prepare food in large quantities, which her children consumed in large amounts.

*“They can eat more if I make it myself. It is costly to purchase. Besides, there is not enough food. There are many people in this household. Since there are many individuals at home, I prepare meals myself. If I buy it, it may be a waste of money. I have to spend a lot of money because it is expensive. Many people can eat a chicken if I cook it myself.”* (Mrs. CY, 27 years, homemaker)

In addition, maternal working status also influenced home food preparation. In this study, two-thirds of the mothers were involved in the labour force. Their working schedules involved long hours, fixed schedules, or shift work, resulting in time constraints related to home food preparation. Therefore, they usually feed their children throughout their hectic schedules by purchasing takeaway food because this option was more convenient than home food preparation. Mrs. KZ, a fish seller, always bought takeaway food because she often arrived home late and did not have enough time to cook food for her family;

*“I do not cook. Sometimes, I have time to cook; sometimes, I do not. I buy food from food outlets. Indeed, I do not have time. I return home after 1 pm. I do not have time.”* (Mrs. KZ, 47 years, fish seller)

Aside from purchasing takeaway food, working mothers also preferred to cook instant food for their children whenever they had limited time to prepare meals. Many mothers said that instant foods, including frozen food and instant noodles, were always available in their kitchens. They usually prepared these whenever they were in a hurry or arrived home late after work because these food products required less preparation time and could quickly satisfy hungry children. As shared by Mrs. KF, a food seller:

*“Every morning, I prepare meals for my children. It is either nuggets or hot dogs. Sometimes, I cook french fries. It is challenging to prepare complete meals. I do not cook fried rice for breakfast.”* (Mrs. KF, 43 years, food seller)

Some mothers experiencing financial hardship claimed that preparing several dishes during mealtime, which required a higher budget, was beyond their means. Thus, they usually cooked one type of dish with fewer ingredients or condiments because it was an economical choice. Even though they did not have variety on their menus, they believed the foods they prepared were delicious because their children often consumed them in large quantities. For instance, Mrs. SZ described that her children ate food in large quantities, even though she served simple dishes, such as fried eggs with sauces, whenever her family had limited financial resources:

*“I occasionally make fried eggs. I know my kid will eat it. He also likes to put soy sauce on his egg. He eats well. Sometimes, he will eat three plates of rice.”* (Mrs. SZ, 44 years, cleaner)

Several mothers experiencing financial constraints described preparing simple meals with limited food variety, indicating that economic hardship influenced both meal composition and dietary diversity within households. This theme revealed that mothers often negotiated food choices within the constraints of financial hardship, time limitations, and their children’s food preferences. Therefore, as these findings indicated, household dietary practices were strongly influenced by broader socioeconomic and household dynamics rather than personal preference alone.

##### Sub-theme 1.3: Skills:

In some households, the mothers were not the only food preparers. They pointed out that their daughters also had good cooking skills and often prepared food for their families. Some of the mothers, such as Mrs. KZ, mentioned that their children were good at cooking fried food and traditional desserts.

*“On Thursdays and Fridays, if she feels like making dinner, she makes dinner. She makes pacts with her friends on occasion, typically when there is a school program. ‘Here, you make this dish, and I will make that.’ She makes fried ‘popiah’ (spring rolls). ‘Popiah’ stuffed with crab filling. She did it on her own.”* (Mrs. KZ, 47 years, fish seller)

The qualitative findings complemented the quantitative results by explaining how maternal education, household economic constraints, and caregiving practices influenced food-related decisions within families. While the quantitative findings demonstrated links between socio-demographic characteristics and obesity risk, qualitative data from the interviews revealed the behavioral mechanisms underlying these relationships, including reliance on takeaway foods, consumption of inexpensive energy-dense foods, and limited time for home food preparation.

#### Theme 2: Social environment.

The second theme from the interviews related to intergenerational OW/OB is the social environment. Under this, two sub-themes were identified: the mother-child relationship and paternal influence, which were factors that determine dietary behavior and physical activities.

##### Sub-theme 2.1: Mother-child relationship:

In most families, the children consumed foods similar to what their mothers ate because the dishes prepared by their mothers were usually identical for all family members. Unintentionally, modelling unhealthy dietary behavior can adversely affect children’s food intake. This pattern was described by many mothers, particularly among households where family members routinely shared the same meals and eating practices. For instance, children developed picky eating behaviors by modelling their mothers’ fussy nutritional habits. Mrs. KN recounted how she was very particular about what she ate and how much she liked fried food. Consequently, her daughter also developed similar food preferences:

*“She is a picky eater too. She imitates her mother in every way. She will not eat rice unless I add it with fried meat or chicken. She will not eat if the food is dry. She consumes the fried meat’s oil.”* (Mrs. KN, 45 years, food seller)

Like dietary behavior, maternal modelling of physical activity can also influence children to be physically active. Few mothers acknowledged the benefits of exercise on body weight management. They described asking their children to be their companions whenever they wanted to exercise at the park or at home. However, some mothers admitted that they were unable to exercise regularly with their children because they had hectic schedules:

*“The child has begun attending school. She had been on a school break before this. So, there was no reason we couldn’t go every day. I brought my children along. We hopped on the bike and rode off. Now, it appears that she has become too busy since returning to school. She is always tired too. Even though I want her to come along, she is too exhausted. So, I choose not to go. It has been a month.”* (Mrs. CY, 27 years, homemaker)

Furthermore, some mothers mentioned that they often offer food to their children to comfort their negative emotions, such as when they felt upset or stressed. Mrs. KF, mother of a non-verbal autistic child, shared her experience in which her son constantly felt hungry even though he had just finished his meal. He continually requested food and would throw a tantrum if he did not get what he wanted. Mrs. KF expressed that she always felt apprehensive whenever her son hurt himself. Therefore, she admitted that she would just serve him food constantly to keep him quiet and calm. According to Mrs. KF;

*“He has eaten fried noodles at 10.30 p.m. After that, he requests rice at midnight. So, I give him rice. Later, at 2.00 a.m., he requests rice because he feels hungry again. If I do not give it to him, he will cause a commotion in the kitchen.”* (Mrs KF, 43 years, homemaker)

Almost all mothers mentioned that they did not consume meals for breakfast together with their children. One of the reasons was that they had limited time in the morning due to occupational and domestic activities. They admitted that they usually skipped breakfast or ate meals at irregular times. Mothers and husbands typically bought takeaway food or ate breakfast at work, while their children at theirs at school. One of the mothers, Mrs. NA, recalled her childhood memories of having meals with her parents. Nonetheless, she could not practice family meals with her children because she perceived that the situation was very different between now and then:

*“There will be no family mealtimes. I eat my meals on my own. I occasionally eat even though I serve the food. During that time, they are still full. In the past, I ate with my parents. Now, it is different. Who is hungry? Eat. Since I am full, I will not eat anything. That is how it is. We eat separately.”* (Mrs NA, 47 years, cleaner)

##### Sub-theme 2.2: Paternal influence:

The children also learned dietary behavior and food preferences from their fathers. Many mothers stated that their husbands preferred to drink sugary beverages daily. Thus, they usually served sugary drinks to their husbands during breakfast, teatime and dinner. When they prepared those drinks for their husbands, their children typically requested same beverages. Therefore, they usually prepared these drinks in large quantities so that their children can have some along with their fathers. As shared by Mrs. KW;

*“Whatever his father makes, he makes them too. He would make iced tea if I make a jug of tea. He adds ice and water. He always requests chilled beverages. He does not want to drink hot water. When I make tea every morning, he will get ice and pour tea in the ice.”* (Mrs. KW, 38 years, homemaker)

In this study, parental modelling emerged not only as a behavioral influence but also as a mechanism through which unhealthy dietary and sedentary practices were passed on and normalized within the family environment and subsequently followed by their children. The findings from both approaches converged in terms of demonstrating the important role of family dynamics in intergenerational obesity. Specifically, in the quantitative approach, maternal and household characteristics were associated with intergenerational obesity risk, while the qualitative findings illustrated how parental modelling, feeding practices, and family routines contributed to the transmission of unhealthy dietary and sedentary behaviors between OW/OB mothers and their children.

#### Theme 3: Physical environment.

The sub-themes related to the theme of the physical environment in intergenerational OW/OB are the home food environment and the built environment.

##### Sub-theme 3.1: Home food environment:

During the interviews, the mothers were asked to describe the types of food available in their homes. Most of them reported having junk food in their homes. They often bought these foods to prevent their children from getting hungry. They always stockpiled biscuits, potato chips, fish crackers, hot dogs, french fries, and instant noodles. Mrs. KL, a food seller, explained that she always purchased and stocked instant food at home every month. Notably, because instant food was easy to prepare and dis not require good cooking skills, she said that her daughter was able to prepare the instant food herself whenever she felt exhausted and lacked the energy to cook for her family. As described by Mrs. KL;

*“I dido purchase them, but only once every month. If they are french fries, I do not always consume them. They are kept in the refrigerator. I am always exhausted when I reach home after work. Who wants to do the cooking anyway? I will not eat unless she (her daughter) prepares the food.”* (Mrs. KL, 47 years, food seller)

The easy availability of junk food at home has influenced mothers and their children to practice unhealthy snacking habits. Because junk food can be consumed at any time, one of the mothers admitted that she always snacksed on junk food while watching television, and the quantity increased at night.

*“I eat at my house. There is everything at home. Even though I have eaten my meal, I often snack while watching television. Perhaps, I eat more at night.”* (Mrs KL, 47 years, food seller)

##### Sub-theme 3.2: Built environment:

The types of food outlets in the neighborhood determine the mothers’ choice of location for purchasing foods. For instance, some mothers preferred to buy food at low-price supermarkets, such as Mydin and Tesco, because they were more economical than grocery shops. Moreover, various brands are readily available at low-cost supermarkets with diverse prices. They also preferred to purchase at low-cost supermarkets rather than groceries because of the price promotions. As described by Mrs. KA, she only purchased discounted foods whenever she had to do grocery shopping:

*“I will buy anything that is on sale, whether they are hotdogs or burger patties. If there is no sale, I will not buy it. I am willing to spend 6 Ringgit if it means I can get three goods. If it costs more than 2 Ringgit for a single unit, I will not purchase it. My children will not purchase expensive goods. We check the price.”* (Mrs. KA, 41 years, homemaker)

Some of the mothers mentioned that they occasionally brought their children to supermarkets. During family shopping trips, children often requested junk foods because they were placed within their line of sight and could be easily reached. The variety of junk foods available in the supermarkets is illustrated in Mrs. KF’s description:

*“There are crackers and toys. The kids also noticed that there were chocolates at the bottom. They took it. Besides, we can find Vitagen and other beverages.”* (Mrs. KF, 43 years, food seller)

Some mothers described that the grocery shops were within walking distance of their homes, while a few others stated that roadside hawker stalls were available in their neighborhoods. Thus, children could purchase unhealthy foods at any time. During her interview, Mrs. SM showed a grocery shop in front of her house. As her daughter was a picky eater, she always gave her daughter pocket money to buy any food she wished to eat. Even though Mrs. SM knew that her daughter often bought soft drinks and potato chips that had high salt and calorie contents, she said she could not control her daughter because various junk foods were available in the stores nearby:

*“She buys potato chips. Potato chips are also said to be salty. Am I correct? Since they like it, what can I do? I give her money and let her buy anything. I am unable to stop it either. There are numerous things (in the shop). It is also said to contain Ajinomoto seasonings, right? I [still]permitted her to make the purchase. At least, she eats something.”* (Mrs. SM, 44 years, cleaner)

Some mothers who were part of this study mentioned that their neighborhoods lacked public amenities, such as parks, playgrounds, or sports facilities. Mrs. KL, who lived in a rural area, commented that her house was far from green spaces. For this reason, her children were not interested in outdoor activities and usually spent more time at home. As Mrs. KL explained:

*“The park is far away. He does not seem to be interested in anything. He does not go anywhere and just uses his mobile phone at home. He stays at home.”* (Mrs. KL, 47 years, food seller)

Meanwhile, some mothers raised safety issues to explain why their children are not involved in outdoor activities. Due to high crime rates in their neighborhoods, one of their concerns was crime-related safety. A few mothers admitted that they did not allow their children to engage in outdoor activities because they worried that they would be exposed to stranger danger. They only allowed their children to do outdoor activities with adult supervision. Mrs. SJ shared her concerns as follows:

*“She rarely goes to the park. Her dad will take her there if he is around. Otherwise, she rarely leaves the house. She rides her bicycle outdoors. It is also the thing that she kept asking for. She would not go if I did not permit her to do so. She leads a sedentary lifestyle. She does not have any friends. She wants to make friends. Sometimes I have a lot of negative thoughts. Particularly in the close-by neighborhoods. There are a lot of naughty boys.”* (Mrs. SJ, 30 years, factory worker)

Apart from above, a few mothers mentioned that they did not allow their children to play outside because of traffic-related safety concerns. As explained by Mrs. SR, her son had to play by the roadsides because there was no recreational park near her residential area. Yet, even though she allowed her son to play outside occasionally, she still worried about his safety. Sometimes, she prevented her son from going outside at all, especially if there were many vehicles on the road.

*“It does not appear to be a suitable location. Some of the areas have many cars passing by. However, this is the only place available to play. That is the only available spot in this location.”* (Mrs. SR, 45 years, homemaker)

While some mothers did mention the availability of public playgrounds in their residential areas, a few complained that the equipment and cleanliness of these green spaces were poorly maintained, rendering them unsafe to be used by the children. Mrs. KF, who lived in a flat house, said that she did not allow her son to play at the playground near her home because the public amenities were broken. She also worried that the broken glasses on the ground could injure her son. Therefore, she only allowed her son to play with his friends within the corridors of the flat house:

*“I do not allow him to go to the park. The park is obviously in terrible shape. There are lots of shattered glasses caused by drunk people.”* (Mrs. KF, 43 years, food seller)

Concerns regarding neighborhood safety and limited recreational facilities were more frequently raised among mothers living in densely populated low-income housing areas. These findings suggest that environmental constraints, including limited access to safe recreational spaces and affordable healthy foods, reinforced sedentary lifestyles and reliance on inexpensive energy-dense foods among both mothers and children.

#### Theme 4: Macro-level environment.

The interviews with the mothers also underscored issues related to the macro-level environment that influences their dietary behavior and physical activities. Two sub-themes emerged from this main theme: food price and media influence.

##### Sub-theme 4.1: Food price:

The results showed that, because of financial hardship, food prices strongly influenced some mothers’ food-purchasing decisions. Concerns regarding increasing food prices were consistently reported across the participants, regardless of employment status, indicating that financial pressure was a shared challenge among low-income households. In particular, the mothers shared that they usually experienced heavy financial burdens, especially for essential needs. Most had to prioritize their house rental fees, utility bills, children’s schooling expenses, and food. They also confessed that they could not afford fruits because they were pricey. Therefore, fruits were rarely available in their homes. For example, Mrs. NK, a mother of three children, confessed that she rarely purchased fruit because of the high cost. She admitted that she came from an impoverished family because she was just a homemaker, while her husband was a village worker who received inconsistent incomes from engaging in agricultural labor. Mrs. NK explained their situation as follows:

*“The cost of fruit is high. We are poor. We rarely purchase fresh fruit. Only coconuts are available (laughs). They do not cost anything because my husband can climb up on the coconut tree by himself to get them. We cannot afford to buy Sunkist oranges. The price is too high for me to pay.”* (Mrs. NK, 38 years, homemaker)

Aside from neglecting expensive healthful foods, some mother often purchased and stockpiled low-cost foods such as anchovies, salted fish, and instant noodles which were always available at their homes as emergency food supplies. Their families consumed these foods whenever they had limited cash for fresh foods.

Many mothers shared that fast foods, such as KFC and McDonald’s, were expensive. Because of their limited financial resources, they always prepared fast foods similar to those sold by Western outlets so as not to frustrate or disappoint their children. They also believed that homemade fast food was a cost-saving option that their children could consume in large quantities until they felt satisfied. In her interview, Mrs. SZ shared her strategy whenever her children requested to eat fried chicken from KFC:

*“Even though I have a job, the income that I have earned is fairly adequate to cover my food expenses. Sometimes, when I want to buy something like that, it is more cost-effective for me to prepare it myself. So, I buy a chicken. Later, I cooked it with KFC flour so it became KFC. It is exactly like that. After that, the sauce is added.”* (Mrs SZ, 44 years, cleaner)

##### Sub-theme 4.2: Media influence:

Almost all families in this study had a television at home, except for two who were unable to buy one. Notably, the availability of television at home affected children’s physical activities. For example, as described by Mrs. KI, her son preferred watching television at home rather than engaging in outdoor activities with his friends:

*“He dislikes interacting with other people. In fact, he has always preferred to spend time at home. He does not have a handphone, thus it is not because of the handphone. He sits and watches television there.”* (Mrs KI, 41 years, cleaner)

Some of the mothers also recounted that when their children used mobile phones, they sleep late and developed the habit of consuming food at midnight. According to Mrs. SW, whenever her son slept late during weekends, she always noticed dirty dishes piled high in the kitchen sink in the morning. She thought that her child always consumed food at midnight while watching videos on the internet:

*“On Saturday and Sunday nights, if he is unable to fall asleep, he stays up and watches videos on YouTube. He eats again at 1 a.m. Then, between 1.30 a.m. and 2:00 a.m., he gets up. He goes back to pick up the plate. I am unsure of the amounts [of food]. I have no idea why there are always so many dirty dishes in the washing bin. Even after washing them all, there are still a lot of dishes. It is because of this fat boy. He eats at 2:00 a.m. He eats at 3:00 a.m. He eats at 4:00 a.m. Later, before he goes to bed, he eats again. That is why he has a big body.”* (Mrs SW, 37 years, homemaker)

A few mothers acknowledged that their children learned to cook new recipes by watching culinary videos on mobile phones. They gave examples of several social media platforms, such as YouTube, Instagram, and Facebook, that helped them get new recipes. Some of the mothers recalled eating, such as cakes, traditional desserts, fried rice, and Western foods, prepared by their children. For example, during her interview, Mrs. KL praised her 17-year-old daughter for making delicious foods. She shared that her daughter not only had talent in cooking international dishes, such as Western foods, which she learned from social media, but she also learned to improvise and modify the recipes to suit her family’s taste:

*“It has a wide variety. It depends on her. She gets a lot of her cooking inspiration from Instagram, for example, food from other countries. She cooks extraordinary food. That is how it is. She prepares spaghetti as well. She also makes macaroni.”* (Mrs KL, 47 years, food seller)

Overall, the qualitative findings expanded the quantitative results by highlighting broader environmental and structural influences that were not captured in the survey data. Factors such as food prices, neighborhood safety, accessibility of recreational spaces, and availability of unhealthy foods emerged as important contextual determinants that may help explain the presence of intergenerational obesity among Malaysian low-income households. Taken together, the integration of quantitative and qualitative findings demonstrated convergence between the socio-demographic patterns identified in the national survey data and the lived experiences described by all the participants.

## Discussion

This study provides an understanding of how intergenerational OW/OB occurs in low-income households, using a mixed methods study design. This mixed methods approach used in this study provided added value by enabling the integration of population-level epidemiological patterns with contextual insights from the participants’ lived experiences. In particular, while the quantitative findings identified socio-demographic factors associated with intergenerational obesity, the qualitative findings helped explain the behavioral, social, environmental, and economic mechanisms underlying these associations within low-income households.

Although a past study has demonstrated the factors associated with intergenerational OW/OB in low-income Malaysian households [20], these factors were limited to the socio-demographic factors regarding mothers and their children. In comparison, the present study contributes to the literature by identifying additional factors beyond the socio-demographic factors that involved in intergenerational OW/OB in low-income households and demonstrating how these factors interact with one another. Furthermore, the integration of both datasets strengthened the interpretation of the findings through triangulation, whereby the qualitative narratives converged with and expanded upon the quantitative associations observed in the national survey data. Therefore, this integration allowed for a more comprehensive understanding of how individual, family, and environmental factors collectively contribute to intergenerational obesity, particularly in low-income households.

Household size may influence obesity-related behaviors through its effects on household food preparation and food purchasing practices. The observed protective influence of having a larger household size may reflect differences in food preparation patterns and household resource allocation among low-income families. A qualitative finding of this study revealed that household size can influence home food preparation. Mothers who lived with many family members preferred food preparation at home rather than buying takeaway food because they perceived the latter as being more expensive than the former, similar to a previous study [33]. In contrast, mothers from small families prefer to buy takeaway food because it is economical. However, individuals who consume takeaway food are more likely to have larger portion sizes and higher energy intakes, coupled with a lack of vegetable and fruit intake [34]. Frequent food intake away from home was also significantly associated with a higher BMI [35]. These findings suggested that frequent food consumption away from home is related to unhealthy dietary behavior and an increased risk of OW/OB among mothers and children.

Next, time constraints related to maternal employment may have led to a greater reliance on convenience and takeaway foods among low-income households. A lack of time to cook food at home encourages working mothers to either purchase takeaway food or cook instant food for their children. However, it has been reported that although instant food can decrease food preparation time, frequent consumption can elevate energy intake due to high-calorie content, resulting in higher energy body weight and BMI [36].

During times of financial constraints, some of the mothers also had to limit their budgets by cooking just one dish every mealtime. Mothers who feed their children by serving only one every meal reported that their children d an imbalanced diet and had poor dietary diversity. This finding supports prior research, which reported that one in five Malaysian households cannot provide various food options to their children and merely depend on inexpensive food [37]. Furthermore, consumption of an imbalanced diet or a lack of variety in food intake can increase the risk of obesity in the future [38].

In the qualitative approach via interviews, many mothers claimed that their children’s food preferences determined the type of food purchased and prepared at home. This finding agrees with a qualitative study of parents from disadvantaged neighborhoods [39]. However, children’s food preferences have also been predominantly linked to unhealthy dietary intake [40]. For example, the mothers in the present study did not prepare vegetable dishes during mealtimes because their children refused to eat them, resulting in inadequate vegetable intake among both the mothers and their children.

Individuals from low-income families believe that wasting food is similar to wasting money [41]. Thus, the mothers included in the present study avoided food wastage is by consuming their children leftovers, similar to parents from low-income families in other country [42]. However, it has been shown that eating consumption leftovers can lead to overconsumption and is associated with increased body weight and BMI [43].

Poor attitudes towards the dietary intake of the mothers and their children were identified in this study. In particular, few mothers reported that they were lazy to prepare home-cooked meals and chose takeaway food instead. In a study investigating dietary behavior, researchers found that consumers often seek convenience opt for convenience, which resulting in the excess purchase of takeaway food [44].

Notably, some of the mothers in this study reported that their children knew how to prepare food and have possessed good cooking skills. A previous study demonstrated that cooking skills were related to healthier dietary behaviors [45]. However, as reported by some mothers in the present study, their children only knew how to cooking energy-dense foods, which might result in poor dietary intake and an increased the risk for OW/OB.

The social environment factor that emerged during the interviews was parental modelling. Some of the the mothers in this study believed that their health-related behaviors influenced their children’s dietary behavior and physical activities through modelling. For instance, their children learned dietary behaviors from their parents during mealtimes. Parental modelling of healthy dietary behavior can help children acquire and practice healthy eating habits [46]. However, the mothers in this study exhibited unhealthy dietary behavior, such as being picky eaters and having a frequent intake of energy-dense foods. Likewise, paternal modelling of unhealthy dietary behaviour, such as consuming sugary beverages, was also perceived to influence children’s food intake in this study, consistent with the findings of previous qualitative studies [47].

Maternal modeling of physical activities can encourage children to be more physically active. When children see their parents engage in any kind of physical activity or exercise, they are more likely to join and do the same activities with their parents. In a previous qualitative study, parents discussed how jointly participating in activities with their children encouraged the latter to do more physical activities [48]. Previous evidence also suggests that physically active parents can decrease the risk of having OW/OB children [49].

During the interviews in the present study, it was noted that some mothers often used food to regulate their children’s emotions, offering them high-calorie foods. In the existing literature, parents who used food to regulate their children’s distress often lead the children to consume more food [50]. During stress, the cortisol hormone is elevated, which, in turn, can cause increased appetite and preference for energy-dense food [51]. Consequently, parents who use food to comfort negative emotions expose their children to a higher risk of being OW/OB in the future [52].

In the present study, family mealtimes during breakfast were rarely practised by most families due to time constraints. The lack of time for family meals due to work constraints and the tight schedules of family members were also quoted by participants a previous study [53]. Although the frequency of family meals was not significantly associated with the risk of OW/OB among adults [54], having such meals with family members can protect children against OW/OB and promote healthy dietary behavior [55].

Most of the mothers interviewed in this study said that while they were always the ones who purchased and stocked foods at home, their children largely determined the types of food purchased, usually junk foods. Some of the mothers also reported that they often purchased inexpensive anchovies, salted fish, and instant noodles due to limited financial resources. However, these foods lack nutrients because they are low-quality protein sources with high sodium and calorie content. The availability of healthy and unhealthy foods at home has been linked to family members’ intake of such foods [56]. In particular, evidence has shown that children who had junk food at home and were allowed to consume it anytime they wanted had significantly high junk food intake [57]. However, the frequent consumption of such low-quality and unhealthy foods can increase the risk of being OW/OB [58].

Many mothers preferred low-cost supermarkets over grocery shops because they offered multiple cheap products and discounted price. This finding is consistent with a previous study, which found that low-income families relied on low prices and special promotions when purchasing groceries [59]. Price promotions, however, are often applied to unhealthy foods and beverages [60]; hence, such promotions encourage consumers to purchase large quantities and stockpile unhealthy foods high in fat, sugar, and salt. Hence, by any chance, the availability of and accessibility to supermarkets in the neighborhoods may possibly increase the risk of obesity.

Easy access to inexpensive energy-dense foods within neighborhood food environments also reinforces unhealthy dietary habits among children. Local fast foods sold at roadside stalls are often high in calories, sugar, and salt [61]. These nutrient-poor and energy-dense foods are also cheaper than healthy foods [62]. Thus, having greater access to such food outlets, coupled with low prices of unhealthy foods encourages more children to buy junk foods. Furthermore, children who receive pocket money from their parents are more likely to consume fast foods, snacks, and sugary drinks and are thus at a higher risk of being OW/OB [63].

In terms of the physical environment, the availability of and accessibility to recreational and sports amenities were associated with higher physical activity levels and reduced sedentary behaviors among children [64]. However, the mothers interviewed in the present study shared that their children did not engage in outdoor activities due to a lack of green spaces and recreational amenities. This finding is similar to that of another qualitative study [65].

Environmental safety concerns may also indirectly contribute to sedentary lifestyles by limiting children’s opportunities for outdoor physical activities. In line with other studies [48,65,66], various aspects of safety were discussed by the mothers in this study, such as concerns regarding neighbourhood crime, heavy traffic, and poor maintenance of public amenities, which also limited children’s physical activities. Related to this, parents who restricted their children from engaging in outdoor activities due to safety concerns may have indirectly influenced the latter’s increased sedentary behaviors, such as watching television and using mobile phones at home.

Another reason for the increased risk of OW/OB among low-income households is the high cost of nutritious foods, which disproportionately affects dietary quality. A previous study showed that the general cost of fruits and vegetables in Malaysia was higher than those of other food groups [67]. However, the strategies adopted by mothers, such as cutting down fruits from their budget due to budget concerns, can lead to inadequate fruit intake and unbalanced diets, which in turn increase adiposity among both adults and children [68].

The increasing accessibility of fast foods may further complicate obesity prevention efforts among financially constrained households. However, fast-food prices are usually unaffordable for individuals coming from low-income Malaysian households [69]. Therefore, the mothers in the present study preferred to cook fast food themselves, which their children could consume in large quantities. However, even though it has been shown that home food preparation is linked to a healthy diet [70], consuming home-prepared food in great amounts can increase the risk of OW/OB among mothers and children.

Excessive screen time can also affect children’s sleeping time. Children who sleep late at night are prone to eat more food at night [71], wake up late in the morning, and skip breakfast altogether [72]. They also tend to consume energy-dense foods, such as ultra-processed foods [73]. In general, sleeping late regularly has been linked to decreased physical activity [74]. In particular, a lack of sleep due to excessive screen time can increase the risk of childhood obesity [75].

Culinary videos on social media can also affect adolescents’ food preferences and influence them to cook and consume the foods they see online [76]. On the one hand, watching videos of healthy food preparation can influence children to choose and consume healthy food [77]. On the other hand, many culinary videos on social media demonstrate unhealthy food preparation [78]. In the present study, the mothers claimed that their children like to watch culinary videos on social media. Thus, it is highly likely that they have been exposed to unhealthy food, especially high calorie foods that children love.

Overall, the themes and sub-themes that emerged in this study were consistent and supported both the socio-ecological model and social cognitive theory. Specifically, the findings suggest that personal factors (i.e., cognitions, demographics, and skills) influenced environmental factors (i.e., social, physical, and macro-level environments). The socio-ecological model and social cognitive theory were pivotal in demonstrating the complexity of the aetiology of OW/OB, which involved various factors at multiple levels.

The findings of this study suggest that broader socio-environmental influences must be considered when developing obesity prevention programs for low-income households. Multiprong interventions to prevent obesity in mothers and children must be developed based on the ”onion” layers portrayed in the socioecological model. Furthermore, current obesity prevention strategies should move beyond approaches that aim to change individual behaviors and incorporate family-oriented and community-based interventions that address broader socio-environmental determinants of obesity.

A convergent mixed method design has never been applied in addressing intergenerational OW/OB problems among mother-child pairs in Malaysia, especially in epidemiological studies. The findings demonstrate the value of integrating quantitative and qualitative approaches in understanding the complex determinants of intergenerational obesity among low-income households. Moreover, this study may also provide opportunities for researchers in other study fields, such as public health, sociology, and economic sectors. The application of both quantitative and qualitative studies can provide a comprehensive understanding of health problems, such as OW/OB, and help establish effective intervention plans.

The findings also provide potential implications for family-oriented prevention programmes. In particular, future programs may be implemented more effectively by focusing on providing nutrition education for both mothers and their children to prevent intergenerational OW/OB. In the Malaysian context, obesity prevention programs should consider cultural food practices, parental influences, and realistic household socioeconomic realities to ensure that interventions are practical, acceptable, and sustainable, especially for low-income families. Additionally, prevention programs should include activities that encourage mothers and their children to be physically active together. In this regard, safe and secure built environments, such as safe walking paths and recreational facilities in neighborhoods should be the major elements to consider in promoting physical activity and exercise.

The findings of this study have important implications for obesity prevention policies and interventions that aim to serve Malaysian low-income households experiencing financial and environmental constraints. These findings suggest that the affordability and accessibility of healthy food may represent important barriers for low-income households, thus requiring further attention in future public health planning and policy discussions.

At the same time, the findings reveal that healthy food options may be limited among low-income households nutritious foods are often less affordable than energy-dense processed foods. Thus, providing subsidies for nutritious food, such as fruits and vegetables, and initiating taxes for unhealthy calorie-dense food may improve access to affordable healthy foods among financially constrained households and encourage them to purchase and consume healthy food at lower prices, ultimately preventing them from eating food with poor diet quality. Indeed, improving the affordability and accessibility of nutritious foods, particularly fruits and vegetables, may help low-income families adopt healthier dietary practices. Therefore, as shown in the examples above, policies such as healthy food subsidies, price control initiatives, and regulation of unhealthy food marketing may be beneficial in reducing reliance on inexpensive energy-dense foods among vulnerable households.

### Strengths and limitations

The main strength of this study is its use of a mixed methods design incorporating quantitative and qualitative approaches, which provide more comprehensive answers to the research questions. Furthermore, the integration of findings from both studies enhanced methodological rigor by enabling triangulation across datasets and providing complementary perspectives on intergenerational obesity among low-income Malaysian households.

To the best of our knowledge, the current study is also the first to apply a convergent mixed methods design to understand the factors influencing intergenerational OW/OB among mother-child pairs in low-income Malaysian households. The quantitative study benefited from a large sample size because it used nationally representative data from three survey years, thereby strenghtening the robustness of the quantitative findings. Meanwhile, the qualitative approach was guided by the socio-ecological model and social cognitive theory, which helped to understand various factors contributing to OW/OB among mother-child pairs.

Despite these findings, several limitations should be considered when interpreting the findings of this study. Given that the quantitative component was cross-sectional and the qualitative component was exploratory in nature, causal relationships cannot be established from the present findings. Although the quantitative component included participants from multiple Malaysian ethnic groups, the qualitative interviews were conducted exclusively with Malay mothers. Therefore, the qualitative findings primarily reflect the cultural, social, and environmental experiences of Malay low-income households and may not fully explain the quantitative associations observed among non-Malay ethnic groups. This ethnic discrepancy may limit the extent of triangulation between datasets and reduce the generalizability of the integrated mixed methods findings across all Malaysian populations. Therefore, future mixed methods studies should include participants from diverse ethnic backgrounds in both components to better understand ethnic-specific determinants and strengthen the cross-ethnic integration of findings.

Next, the qualitative study participants were recruited using snowball sampling, which may have introduced selection bias and limited the diversity of perspectives represented in the study. Additionally, the qualitative interviews were conducted only among mothers to explore maternal perspectives on intergenerational obesity, which cannot fully capture the views and experiences of children or other family members. Therefore, future studies should consider incorporating parents’ and children’s perspectives to provide a more comprehensive understanding of intergenerational obesity within households.

Furthermore, the qualitative study was designed to explore shared experiences across participants and was not intended to make comparisons between participant subgroups in terms of urban and rural residence, employment status, or household characteristics. Hence, future qualitative studies can use comparative sampling designs to provide a deeper understanding of subgroup-specific differences in intergenerational overweight/obesity experiences.

## Conclusion

Intergenerational OW/OB among mother-child pairs in impoverished households is a significant public health concern in Malaysia. In this study, the integration of quantitative and qualitative findings provided a more comprehensive understanding of the factors contributing to intergenerational OW/OB among mother-child pairs in these low-income households.

The qualitative study complemented the quantitative study by providing information on intergenerational OW/OB in that was not captured in the latter. In particular, children’s preference for high-sugar and high-calorie foods contributes to the availability of such energy-dense foods at home. Living with financial hardship has also led mothers not only to prioritize their children’s food preferences but also to choose to eat leftovers to avoid food waste. Furthermore, mothers in small households and those who were part of the workforce rarely prepared meals at home and preferred to purchase takeaway food. They also often dealt with limited financial resources by purchasing low-cost high-calorie foods and preparing imbalanced meals.

This study also found that parent-child relationships may also contribute to intergenerational OW/OB by modeling unhealthy food intake, feeding practices, and normalizing lack of family mealtimes. Other factors have also been found to influence intergenerational OW/OB through poor dietary habits, due to the availability and accessibility of energy-dense food at home and at food outlets outside the home, and increased sedentary behaviors and reduced physical activities of mother–child pairs due to the lack of available public amenities, and lingering safety issues.

Finally, the study’s findings highlight the need for multilevel and family-oriented obesity prevention strategies that address behavioral and environmental determinants within low-income households. Applying quantitative and qualitative approaches helps in gaining a more comprehensive understanding of how intergenerational OW/OB occurs among mothers and children, especially in low-income households.

## Supporting information

S1 Table Univariable logistic regression for the factors associated with OWM/OWC from low-income households in Malaysia for years 2006, 2011 and 2015. (DOCX)
