# Supplementary material for: Obese mother, obese child: A convergent mixed methods study of intergenerational obesity in low-income households in Malaysia
Source: PLoS One. 2026 Aug 11;21(8):e0355312. doi: 10.1371/journal.pone.0355312 (PMC13460589; doi:10.1371/journal.pone.0355312)
Supplement: Supplementary file 1 [file pone.0355312.s001.docx]

Table S1.	Univariable logistic regression for the factors associated with OWM/OWC from low-income households in Malaysia for years 2006, 2011 and 2015

| Risk factors | | 2006 (n=860) | | | | 2011 (n=384) | | | | 2015 (n=418) | | | |
| --- | --- | --- | --- | --- | --- | --- | --- | --- | --- | --- | --- | --- | --- |
|  |  | OR | 95% CI | | p-value | OR | 95% CI | | p-value | OR | 95% CI | | p-value |
| Maternal age | |  |  |  |  |  |  |  |  |  |  |  |  |
|  | ≤ 30 | 1.00 |  |  |  | 1.00 |  |  |  | 1.00 |  |  |  |
|  | 31 – 40 | **2.11** | **1.12, 3.98** | | **0.021** | 1.10 | 0.49, 2.45 | | 0.818 | 1.48 | 0.71, 3.09 | | 0.301 |
|  | 41 – 50 | **3.99** | **2.13, 7.45** | | **<0.001** | 1.35 | 0.62, 2.98 | | 0.452 | 1.95 | 0.94, 4.06 | | 0.074 |
|  | 51 and above | **4.91** | **2.45, 9.85** | | **<0.001** | 2.26 | 0.91, 5.65 | | 0.081 | **3.17** | **1.37, 7.33** | | **0.007** |
| Child age | |  |  |  |  |  |  |  |  |  |  |  |  |
|  | 5 – 9 | 1.00 |  |  |  | 1.00 |  |  |  | 1.00 |  |  |  |
|  | 10 – 14 | **3.31** | **2.37, 4.63** | | **<0.001** | **2.06** | **1.31, 3.24** | | **0.002** | **1.79** | **1.15, 2.79** | | **0.010** |
|  | 15 – 17 | **2.62** | **1.62, 4.23** | | **<0.001** | 1.33 | 0.70, 2.54 | | 0.388 | 1.00 | 0.58, 1.72 | | 0.993 |
| Child gender | |  |  | |  |  |  | |  |  |  | |  |
|  | Girl | 1.00 |  | |  | 1.00 |  | |  | 1.00 |  | |  |
|  | Boy | 0.88 | 0.66, 1.19 | | 0.416 | 0.93 | 0.62, 1.40 | | 0.594 | 1.27 | 0.86, 1.86 | | 0.231 |
| Household sizeª | |  |  |  |  |  |  |  |  |  |  |  |  |
|  | Small | 1.00 |  |  |  | 1.00 |  |  |  | 1.00 |  |  |  |
|  | Medium | **0.60** | **0.44, 0.83** | | **0.002** | **0.60** | **0.39, 0.91** | | **0.017** | 0.92 | 0.62, 1.37 | | 0.691 |
|  | Large | **0.44** | **0.25, 0.77** | | **0.004** | 0.45 | 0.18, 1.12 | | 0.085 | **0.19** | **0.04, 0.87** | | **0.033** |
| Ethnicity | |  |  |  |  |  |  |  |  |  |  |  |  |
|  | Malay | 1.00 |  |  |  | 1.00 |  |  |  | 1.00 |  |  |  |
|  | Chinese | 0.54 | 0.28, 1.05 | | 0.067 | 0.47 | 0.22, 1.03 | | 0.058 | **0.39** | **0.20, 0.77** | | **0.006** |
|  | Indian | 1.58 | 0.80, 3.10 | | 0.186 | 2.19 | 0.93, 5.15 | | 0.073 | 1.11 | 0.53, 2.33 | | 0.790 |
|  | Other^b^ | **0.41** | **0.28, 0.61** | | **<0.001** | **0.39** | **0.23, 0.68** | | **0.001** | **0.48** | **0.30, 0.77** | | **0.002** |
| Maternal education | |  |  |  |  |  |  |  |  |  |  |  |  |
|  | Tertiary | 1.00 |  |  |  | 1.00 |  |  |  | 1.00 |  |  |  |
|  | Secondary | 0.71 | 0.06, 7.86 | | 0.776 | 0.70 | 0.28, 1.76 | | 0.452 | 1.48 | 0.71, 3.10 | | 0.296 |
|  | Primary | 1.08 | 0.10, 12.02 | | 0.951 | 0.57 | 0.22, 1.50 | | 0.257 | 1.42 | 0.65, 3.08 | | 0.380 |
|  | None | 0.37 | 0.03, 4.19 | | 0.419 | 0.50 | 0.17, 1.50 | | 0.217 | 1.85 | 0.68, 5.00 | | 0.228 |
| Family structure | |  |  | |  |  |  | |  |  |  | |  |
|  | Dual-parent family | 1.00 |  | |  | 1.00 |  | |  | 1.00 |  | |  |
|  | Single-parent family | 0.80 | 0.47, 1.38 | | 0.423 | 1.44 | 0.74, 2.77 | | 0.281 | **2.11** | **1.14, 3.90** | | **0.018** |
| Residential area | |  |  | |  |  |  | |  |  |  | |  |
|  | Rural | 1.00 |  | |  | 1.00 |  | |  | 1.00 |  | |  |
|  | Urban | 1.12 | 0.82, 1.52 | | 0.480 | 1.03 | 0.69, 1.56 | | 0.879 | 0.78 | 0.53, 1.14 | | 0.198 |

*Note.* CI=confidence interval; OR=odds ratio

Statistically significant (p-value<0.05) are highlighted in bold

ª Small=less than five persons; medium=5 to 7 persons; large=more than seven persons in the household

^b^ Other=Other three major ethnic groups in Malaysia, including Bumiputras in Sabah and Sarawak
